# Death Following the Use of Ether

**Published:** 1876-10

**Authors:** 


Death Following the Use of Ether.—A case of cerebral hemorrhage from laceration of blood-vessels, is found in a Boston journal, by Dr. Arthur Mathewson, of Brooklyn, New York, as the result of etherization in the operation of iridectomy. He says: "The operation was brief and the ether was at once removed, having been inhaled less than five minutes. * * * As no unusual symptoms occurred, and as the patient was apparently doing well, she was left with an attendant. Fifteen minutes later Dr. Cornwell, the House Surgeon, was called to see the patient, the attendant saying she had a 'fit,' and reported that five minutes after we had left her there had been several ineffectual efforts to vomit, which were followed in a 'couple of minutes' by a 'fit.' Dr. Cornwell found her cyanotic, breathing stertorously, bathed in profuse perspiration, and unconscious. The pulse was 120 per minute, soft, and compressible. I saw her at 5:30 (one hour after the operation), when she was in much the same condition, presenting the symptoms of cerebral hemorrhage. * * * On removal of the calvaria a large hemorrhagic patch was found under the arachnoid, on the upper surface of the middle portion of the left hemisphere."

This case seems to have been reported as one of death from the direct result of anæsthesia by ether. Death from the direct effects of anæsthetics—chloroform and ether—occurs always, so far as we are informed, before the patient revives from stupor. As "the patient was apparently doing well" when the operator left her, she had certainly recovered from the anæsthesia, and from the nurse's report of frequent attempts at vomiting before having the "fit," the inference is that nausea, which often follows the use of ether, in this case resulted in retching, and the effort to vomit ruptured the cerebral blood-vessels. This result might have occurred from vomiting caused by any other means, in the unhealthy state of the blood-vessels, which probably existed in this case.

